# A cross-sectional analysis of the relationships between anxiety sensitivity and youth irritability: the mediated roles of insomnia and selective attention for threat

**DOI:** 10.1186/s12888-023-05280-z

**Published:** 2023-10-25

**Authors:** Yalin Li, Wanfu Tian, Ping Liu, Fulei Geng

**Affiliations:** 1 School of Psychology, Jiangxi Normal University, 99 Ziyang Ave, Nanchang, Jiangxi 330022 China; 2 Chenzhou Xiangnan Middle School, Chenzhou, China

**Keywords:** Irritability, Anxiety sensitivity, Insomnia, Selective attention for threat, Youth, Reward

## Abstract

**Background:**

Irritability is common in multiple psychiatric disorders and is hallmark of disruptive mood dysregulation disorder. Child irritability is associated with higher risk of suicide and adulthood mental health problems. However, the psychological mechanisms of irritability are understudied. This study examined the relationship between anxiety sensitivity and irritability among youth, and further explored three possible mediated factors: selective attention for threat, delayed reward discounting, and insomnia.

**Methods:**

Participants were 1417 students (51.7% male; mean age 13.83 years, SD = 1.48) recruited from one high school in Hunan province, China. Self-report questionnaires were used to measure irritability (The Affective Reactivity Index and The Brief Irritability Test), anxiety sensitivity (The Childhood Anxiety Sensitivity Index), selective attention for threat (The Davos Assessment of Cognitive Biases Scale-attention for threat bias subscale), insomnia (The Youth Self-Rating Insomnia Scale), and delayed reward discounting (The 27-item Monetary Choice Questionnaire). Structural equation modal (SEM) was performed to examine mediated relations.

**Results:**

Anxiety sensitivity was modestly related to irritability and insomnia (r from 0.25 to 0.54) and slightly correlated with selective attention for threat (r from 0.12 to 0.28). However, there is no significant relationship of delayed rewards discounting with anxiety sensitivity and irritability. The results of SEM showed that selective attention for threat (indirect effect estimate = 0.04) and insomnia (indirect effect estimate = 0.20) partially mediate the relationship between anxiety sensitivity and irritability, which explained 34% variation.

**Conclusions:**

Anxiety sensitivity is an important susceptibility factor for irritability. Selective attention for threat and insomnia are two mediated mechanisms to understand the relationship between anxiety sensitivity and irritability.

## Introduction

Irritability is a common phenomenon in children and adolescence [1] and is also one of the main reasons for seeking psychiatric treatment in youth [2]. It is defined as a state of proneness to anger, specifically, having lower threshold and more intense response to frustration. Children and adolescents with irritability are characterized by frequent, intense, and disproportionate outbursts of temper and anger [3]. Although irritable symptoms decline with age, a considerable proportion of teens showed significant irritability [4]. Irritability is associated with multiple mental disorders, such as autism spectrum disorders, oppositional defiant disorder, attention deficit hyperactivity disorder, anxiety disorder, major depressive disorder and posttraumatic stress disorder [5–9]. Pathological irritability, that is chronic and persistent anger, is the core feature of disruptive mood dysregulation disorder. Without effective treatment, pediatric irritability could have long-term adverse effects. Two recent review studies indicate that irritability is one very important indicator of suicide [10, 11]. Several longitudinal studies found that childhood irritability was associated with increased mental health problems in adulthood [12].

Despite the clinical significance of irritability, research into the etiological and pathophysiological mechanisms of this symptom is in its nascent stages. Behavioral genetic studies suggest that irritable mood appears to be moderately heritable and is influenced by unique environmental events [2]. This study aimed to examine the relationship between anxiety sensitivity and irritability among youth in a cross-sectional design, and further explore the possibilities of insomnia, selective attention for threat, and delayed reward discounting as mediators. Below, we will briefly review related literature.

## Irritability and anxiety sensitivity

Youth with some personality traits (e.g., nervousness) tend to be at higher risk of irritability [13]. Anxiety sensitivity (AS) is a personality trait and characterized by fear that feelings and symptoms associated with anxiety could yield harmful physical or social consequences. According to the “fear expectation model”, it is defined as “fear from fear” or “fear from anxiety” [14], including physical concerns (e.g., fear of a heart attack from a racing heart), cognitive concerns (e.g., fear of “going crazy” because of difficulty concentrating) and social concerns (e.g., fear of being embarrassed by trembling in public) [15]. As an important susceptibility factor of affective disorders, AS has been reported to be association with major depression disorder, generalized anxiety disorder, post-traumatic stress disorder and obsessive-compulsive disorder [16]. Irritability is one of the diagnostic criteria for multiple affective disorders, and is highly correlated with depression disorder, anxiety disorder, and post-traumatic stress disorder [1]. Thus, common risk factors, such as AS, may be shared between irritability and affective disorders. However, to our best knowledge, the relationship between AS and irritability has yet been examined.

### Irritability and insomnia

Sleep deprivation has identified as important environmental factors for irritability [17]. An experimental research on sleep has shown that lack of sleep has a causal effect on emotional regulation and irritability [18]. Insomnia is an important reason for lack of sleep, and individuals with symptoms of insomnia often experience sleep-related cognitive, emotional, and physical hyperarousal. Dysfunctional emotional responses may be linked to sleep-related hyperarousal [19]. When sleep is restricted, teens are unable to regulate their emotional responses properly, leading to temper outbursts and exaggerated responses to small triggers [20].

### Irritability and the processing of threat and reward

Given that irritability is a trans-diagnostic symptom, researchers generally use Research Domain Criteria (RDoC) strategy to explore its pathological mechanism. Aberrant processing of fear and reward are two more studied lines. Neurophysiology and behavioral studies have shown that individuals with irritability or severe mood dysregulation are more easier to interpret obscure faces as threatening [21, 22] and more likely to turn their attention to threatening faces, relative to neutral faces [23]. Enhanced attention to threat, as well as, selective attention to hostile or threat-related information would inversely contribute to increased levels of anger [24]. Frustration is central to irritability, and irritable youth may be particularly vulnerable to frustration as a result of having impairments in reward processing. Children and adolescents with irritability show significant deficits in learning to reward in the face of unexpected events and a heightened sensitivity to the receipt of rewards and tend to be inclined towards more timely rewards [25, 26]. Irritable youth have a propensity to choose smaller immediate rather than larger delayed rewards [27]. In turn, delayed reward discounting reflects a form of dysfunction seen across externalizing and internalizing psychiatric conditions that may increases risks for irritability [27]. Rewards and threats are not independent of each other and in the context of threats, the processing of rewards also changes, including increased expressions of anger at frustration [2].

### Anxiety sensitivity, insomnia and the processing of threat and reward

Anxiety sensitivity amplifies attention to physiological sensations associated with fear, as well as particular sensitivity to external stimuli that may trigger anxiety, which may explain the bias in threat information processing [28]. Several studies provide evidence that individuals with high anxiety sensitivity prioritize threat information [29, 30]. For example, individuals with high levels of physical AS showed higher vigilance for stimuli associated with anxiety symptomatology compared to neutral words [31]. Recent studies indicated that AS was associated with increased risks of addiction-related problems [32]. Delayed reward discounting is one core feature of individuals with various addictive behaviors. Probably, AS may be related to delayed reward discounting. In addition, anxiety sensitivity is an important risk factor of insomnia severity [33]. Rumination about negative events, excessive worry about future events, and cognitive intrusions, which are frequent and disturbing amongst people on anxiety sensitivity, can hinder the onset of sleep in the pre-sleep period [34].

### Insomnia and processing of threat and reward

A key feature of sleep is the necessary loss of awareness and responsiveness to environmental cues, and this state that is attenuated by excessive focus on threats [35, 36]. As an adaptive function, alertness and mobilization will be promoted in threatening environmental conditions [36]. Insomnia may be associated with a propensity to display vigilant attention to threat. Additionally, some evidence suggests a link between sleep disturbance and reward processing deficits [37, 38]. For example, fewer minutes asleep, later sleep onset time, and lower sleep quality were related to hypoactivation in reward system circuitry during anticipation of rewards [37]. Insomnia has been correlated with decreased effort expended for rewards [39].

### Summary

**Fig. 1:**
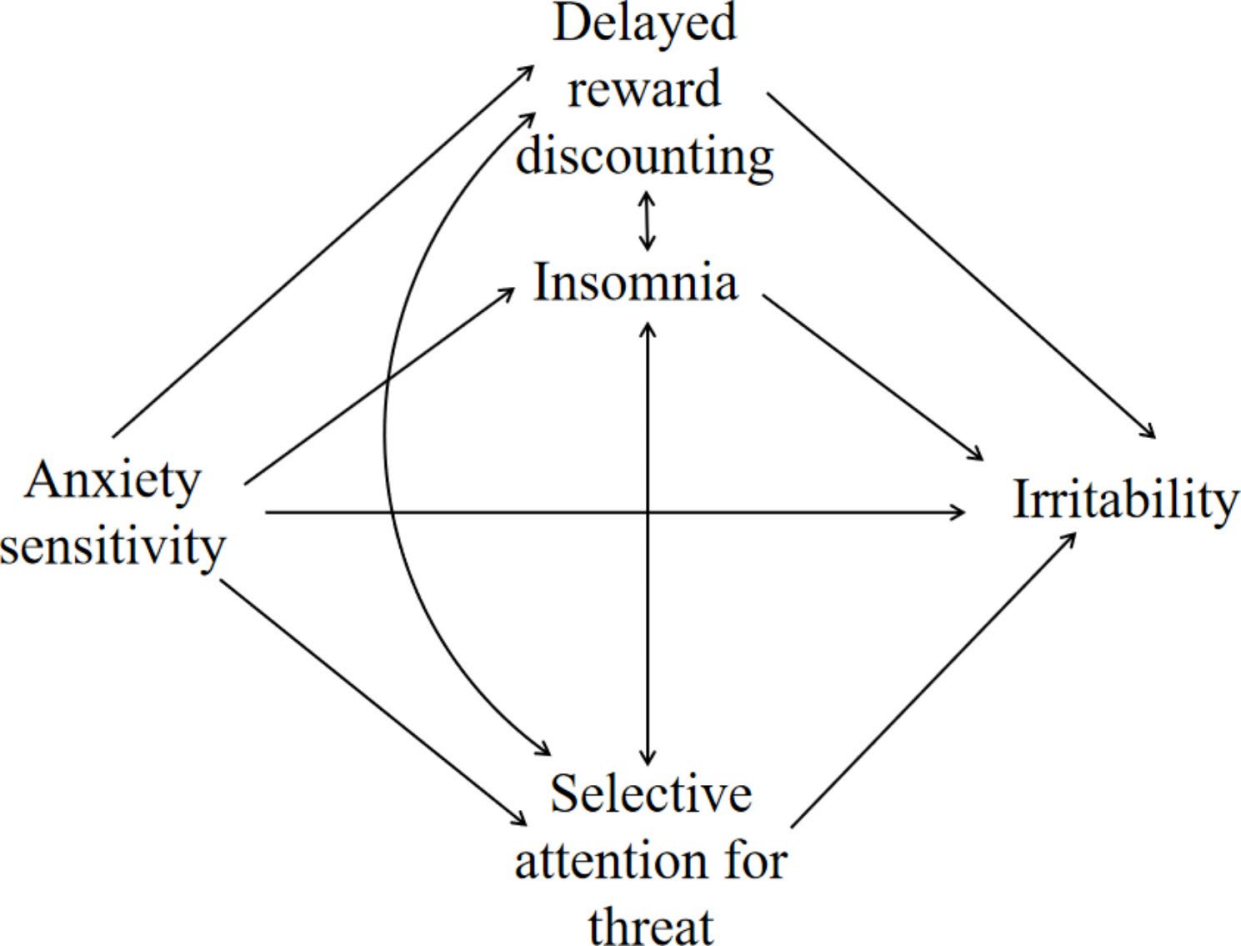
Initial hypothetical model

Irritability is one significant clinical problem that should be elucidated. AS may be one important predisposing factor of irritability, but this possibility has yet been examined. As reviewed above, both irritability and AS are linked to abnormal processing of fear and reward, and AS may increase people’s sleep disturbance and then cause irritable symptoms. Thus, selective attention for threat, delayed reward discounting , and insomnia are three candidate mechanisms to explain the effects of AS on irritability, see Fig. 1. In the present study, we examined the hypothetical model in a sample of Chinese youth.

## Method

### Participants and procedure

Data are derived from one ongoing longitudinal study of psychological mechanisms of irritability among adolescents. Participants were Grade seven and Grade ten students from one middle high school in Chenzhou City, Hunan Province, China. A total of 1600 self-report questionnaires were distributed to target students, 183 of students refused to answer this questionnaire. Finally, 1417 valid data were obtained with a response rate of 88.6%. The mean age of these participants was 13.83 years (SD = 1.48; range from 11 to 17); 51.7% (n = 732) were male; 42.4% (n = 601) were Grade seven students.

The survey was conducted between October 25 and November 22 in 2022. Participants were invited to complete the survey during school days with group format. Two postgraduate students majored in clinical psychology introduced the purposes of the research and answered students’ questions in classrooms. The whole survey took about 30–35 min.

### Measures

#### Anxiety sensitivity

The Childhood Anxiety Sensitivity Index (CASI) is a 18-item self-report measure that can be used to assess the fear of anxious arousal [40]. It comprises three subscales, including physical concerns (e.g., “It scares me when I feel like I am going to faint”), cognitive concerns (e.g., “It scares me when I am unable to keep my mind on a task”) and social concerns (e.g., “It scares me when I blush in front of other people”). Items are rated on a 3-ponit scale from 0 for “None” to 2 for “A lot”. The CASI total score ranges from 0 to 36, with high score indicating more severity of anxiety sensitivity. The CASI has demonstrated great reliability and validity in Chinese children and adolescents [41]. In the present study, the Cronbach’s α was 0.89.

#### Irritability

The Affective Reactivity Index (ARI) is used to measure irritability of self-report in previous six months [42]. It comprises seven items and the seventh item is functional impairment. The ARI is a three-point scale, ranged from 0 for “not true” to 2 for “certainly true”. The sum of scores of first six items was used in the current study. In the Chinese population, the reliability and validity of ARI were great [43]. In the present study, the Cronbach’s α was 0.84.

Brief Irritability Test (BITe) is a 5-item subjective assessment of irritability [44]. Each item is rated on 6-point Likert scale, ranged from 0 for “never” to 5 for “always”. The total scores of BITe range from 0 to 25. The BITe has been reported the excellent reliability and validity [44]. The Chinese version of BITe has been used in one previous study [45]. The Cronbach’s α was 0.85 in the current study.

#### Insomnia

The Youth Self-Rating Insomnia Scale (YSIS) is a 8-item measure designed to assess the insomnia in the past two weeks [46]. The YSIS comprises two factors which are daytime impairment and insomnia assessed eight symptoms (e.g., difficulty in initiating sleep, difficulty in maintaining sleep, early morning awakening and so on). The items are rated on a 5-point scale and the total score of the YSIS ranged from 8 to 40. The cutoffs for insomnia severity are: normal (< 22), mild insomnia (22–25), moderate insomnia (26–29), and severe insomnia (≥ 30) [46]. The Cronbach’s α was 0.80 in the current study.

#### Selective attention for threat

The Davos Assessment of Cognitive Biases Scale (DACOBS) attention for threat bias subscale was used to measure students’ propensity to selective attention for threat [47]. Four items from the Chinese version subscale were included in this study: “I pay more attention to detail in terms of big picture”, “I want to make sure every window is locked”, “To protect myself, I stayed alert” and “I avoid considering information that shakes my opinion” Each term contains seven options, using a scale of 1–7. High score indicates stronger attention for threat bias. The Chinese version of DACOBS has good reliability and validity [48]. The Cronbach’s α was 0.64 in the present study.

#### Delayed reward discounting

The 27-item Monetary Choice Questionnaire was used to measure delayed reward discounting to choose between small rewards available immediately or larger rewards available after a delay [49]. Delayed rewards are grouped into 3 categories (small, medium, and large) based on size, with 9 items per category. The immediate choice ratios (ICR) of MCQ were calculated by using a specific syntax [50]. Higher ICR indicates lower level of delayed reward discounting. In the current study, the Cronbach’s α was 0.94.

### Statistical analyses

Pearson correlation was used to explore the associations of AS with other mediating variables and outcome variables. To keep the model more concise and easier to understand, only significant associations were used in the following analyses. Structural equation model (SEM) was used to examine our hypothetical model. First, measurement models were evaluated using the maximum likelihood (ML). All our core concepts would be modeled as latent variables. The hypothesized indicators are as follows: the CASI three subscale total scores are indicators of AS; the four items from the DACOBS attention for threat bias subscale are indicators of selective attention for threat; the YSIS two subscale total scores are indicators of insomnia; the three ICRs of MCQ are indicators of delayed reward discounting, and the ARI and BITe total scores are two indictors of irritability. Finally, a structural equation model was established to test the psychological mechanisms from AS to irritability with sex and age as covariates for irritability. The model was performed using ML method, and indirect effects were calculated using bootstrap method with 5000 resampling. Model fitting were evaluated using comparative fit index (CFI; > 0.90), Tucker-Lewis index (TLI; > 0.90), the root-mean-square error of approximation (RMSEA; < 0.08), and standardized root mean square residual (SRMR; <0.05/0.08) [51]. Statistical analyses were conducted by using the SPSS21.0 software and the Mplus 8.3 software. In the current sample, the percentage of missing data is pretty low (< 0.5%), thus missing data were dealt with default setting in software, with listwise deletion for SPSS and ML for Mplus.

## Result

### Preliminary analyses

In the current sample, the prevalence rates of mild, moderate, and severe insomnia were 22.6%, 12.8%, and 7.4%, respectively. The irritability was relatively common, as the means of ARI (range from 0 to 12) and BITe (range from 0 to 25) were 1.65 and 6.01, respectively. The details of descriptive statistics and results of Pearson correlations are presented in Table 1. The three dimensions of AS were positively related to irritability, generally the effect sizes are modest. Insomnia and selective attention for threat were significantly related to both AS and irritability, but the magnitudes are pretty low in selective attention for threat. However, there was no significant relations of delayed reward discounting with both AS and irritability.

**Table 1:** Means, SD, and correlations of studied variables

	1	2	3	4	5	6	7	8	9	10	11	12	13	14
1CASI-physical concerns	1.00													
2CASI-cognitive concerns	0.61^**^	1.00												
3CASI-social concerns	0.46^**^	0.37^**^	1.00											
4YSIS-insomnia symptoms	0.29^**^	0.27^**^	0.31^**^	1.00										
5YSIS-daytime impairment	0.33^**^	0.28^**^	0.35^**^	0.46^**^	1.00									
6YSIS-total scores	0.36**	0.32**	0.38**	0.76**	0.92**	1.00								
7ARI	0.40^**^	0.32^**^	0.25^**^	0.27^**^	0.31^**^	0.34**	1.00							
8BITe	0.54^**^	0.50^**^	0.45^**^	0.39^**^	0.44^**^	0.49**	0.64^**^	1.00						
9DACOBS-attention for threat bias	0.25^**^	0.19^**^	0.27^**^	0.13^**^	0.12^**^	0.14**	0.19^**^	0.28^**^	1.00					
10MCQ-small money ICR	− 0.01	− 0.03	− 0.02	− 0.03	0.02	− 0.00	0.06^*^	0.04	− 0.04	1.00				
11MCQ-medium money ICR	− 0.01	− 0.04	− 0.05	− 0.04	0.02	− 0.01	0.05	0.03	− 0.03	0.89^**^	1.00			
12MCQ-large money ICR	− 0.03	− 0.05	− 0.08^**^	− 0.05	− 0.01	− 0.03	0.03	− 0.00	− 0.02	0.84^**^	0.90^**^	1.00		
13sex	0.25^**^	0.16^**^	0.11^**^	0.04	0.18^**^	0.15**	0.16^**^	0.19^**^	0.07^**^	− 0.02	− 0.04	− 0.04	1.00	
14age	0.02	0.05^**^	0.20^**^	0.01	0.15^**^	0.12**	− 0.04	0.08^**^	0.03	0.07^**^	0.06^*^	0.04	− 0.02	1.00
M	5.74	0.67	3.01	6.30	13.96	20.26	1.65	6.01	15.85	0.69	0.63	0.56	1.48	13.83
SD	5.08	1.24	1.78	2.756	4.68	6.36	2.33	5.18	5.06	0.27	0.27	0.27	0.50	1.48

Note: CASI = childhood anxiety sensitivity index; YSIS = youth selfrating insomnia scale; ARI = affective reaction index; BITe = brief irritability test; DACOBS = Davos Assessment of Cognitive Biases Scale; MCQ = monetary choice questionnaire; ICR = immediate choice ratios; sex (male = 1, female = 2); **, p < 0.01, *, p < 0.05

### SEM analyses

**Fig. 2:**
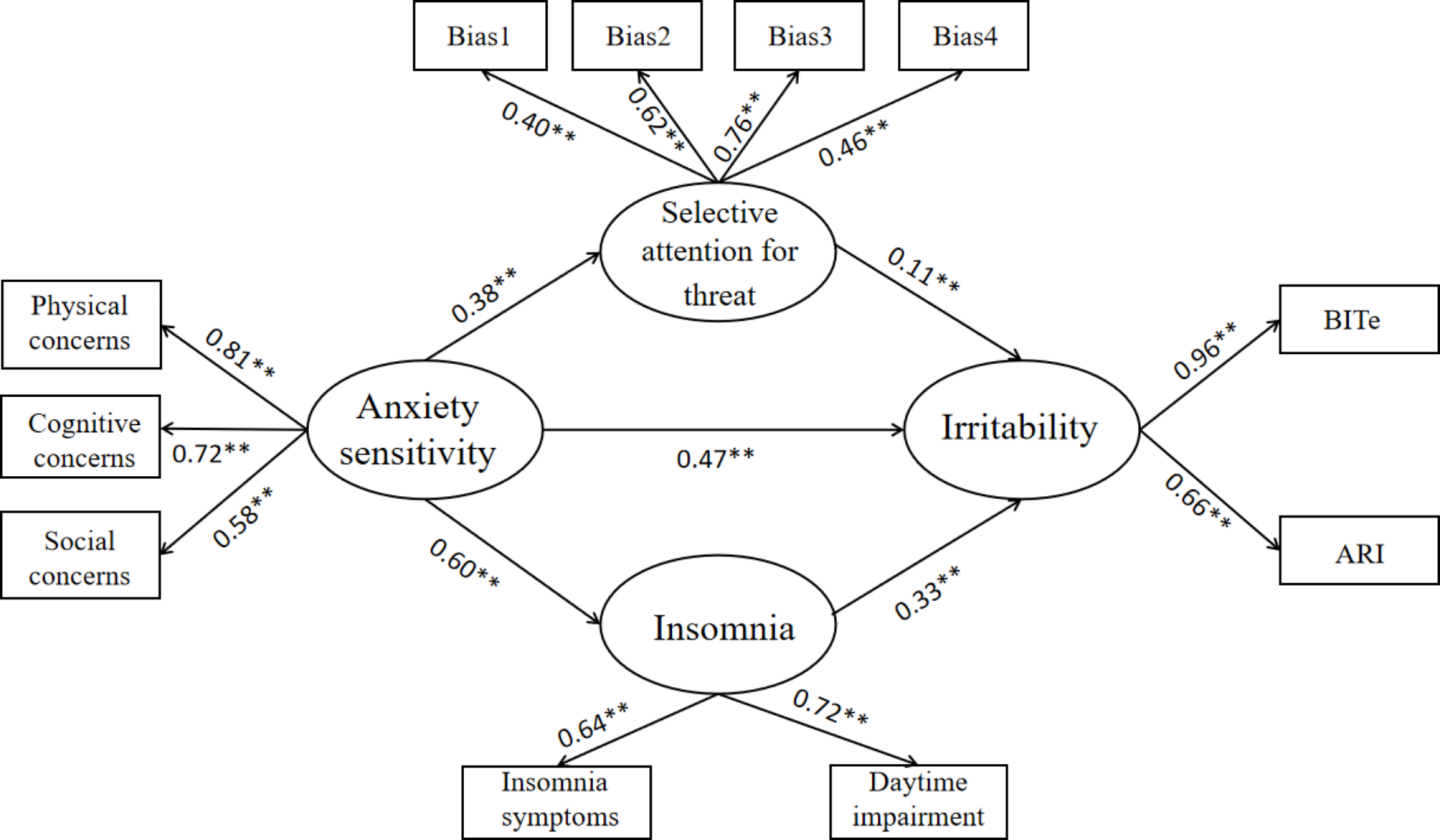
Final structural equation model. Note: BITe = brief irritability test; ARI = affective reaction index

Given that delayed reward discounting was not related to other variables, it was excluded in the following models. The measurement model included AS, insomnia, selective attention for threat, and irritability. The model fit the data pretty well, χ^2^(38) = 155.082, CFI = 0.972, TLI = 0.959, RESEA = 0.047, SRMR = 0.03. All the indicators were loaded on hypothesized latent variables, and the factor loading were all greater than 0.4, although some loadings in the items of selective attention for threat were relatively low. Then, one SEM model, shown in Fig. 2, was evaluated to examine the indirect effects of AS to irritability via insomnia and selective attention for threat. The model also fit the data well, χ^2^(59) = 389.025, CFI = 0.924, TLI = 0.901, RESEA = 0.063, SRMR = 0.057.

The results of bootstrap showed that both the two indirect effects were significant, the estimated effect was higher for insomnia (0.20) and lower for selective attention for threat (0.04). The direct effect from AS to irritability was still significantly after controlling for the above indirect effects, indicating that insomnia and selective attention for threat played partial mediations, see Table 2.

**Table 2:** Standardized direct and indirect pathway of the model

Model pathways	β	*p*	95% CI
**Direct effect**			
Anxiety sensitivity → Irritability	0.466	< 0.001	[0.380, 0.555]
Anxiety sensitivity → Selective attention for threat	0.381	< 0.001	[0.320, 0.445]
Anxiety sensitivity → Insomnia	0.596	< 0.001	[0.530, 0.661]
Selective attention for threat → Irritability	0.112	< 0.001	[0.056, 0.167]
Insomnia → irritability	0.329	< 0.001	[0.239, 0.415]
**Indirect effect**			
Anxiety sensitivity → Selective attention for threat →irritability	0.0423	< 0.001	[0.021, 0.064]
Anxiety sensitivity → Insomnia → irritability	0.196	< 0.001	[0.142, 0.252]

## Discussion

### Anxiety sensitivity and irritability

This study explored the etiological and pathological mechanisms of irritability in a sample of Chinese youth with cross-sectional design. The findings suggest that AS is an important susceptibility factor for irritability. AS has been extensively studied in various affective disorders, such as general anxiety disorder, major depressive disorder and posttraumatic stress disorder [52–54]. Generally, AS is considered as an important transdiagnostic risk factor [55]. Extending previous studies, this study firstly found strong relationship between AS and irritability. This opens up new possibilities to elucidate and intervene irritability, as studies have demonstrated that AS is a key change element in the treatment of pathological anxiety [56]. Both AS and irritability can be deconstructed into different components. Our findings showed that AS may be more tightly linked to emotional component of irritability. Compared to the ARI scale, the BITe is designed to measure emotional component [44] and got the highest correlations among all three dimensions of AS. Additionally, the findings suggest that physical concern is more closely associated with irritability than cognitive concerns and social concerns. One previous study reported that only cognitive concerns was associated with depression [57]. Together, these findings imply that the mechanisms from AS to various affective disorders might be different.

### Delayed reward discounting

Following the RDoC strategy, we further examined whether delayed reward discounting, insomnia, and selective attention for threat played mediating roles between AS and irritability. It is a little surprised that delayed reward discounting was not significantly related both AS and irritability. Considerable research has reported abnormal reward processing and difficulties of impulse control in irritable youth [58, 59]. Specifically, several studies demonstrated that irritability is associated with alterations during multiple reward processing phases, including reward anticipation, reward outcome, and frustrative non-reward [60]. Furthermore, individual traits (e.g., executive function) may buffer irritability-related reward processing deficits [61]. In a sample of adolescents with conduct disorder, one recent study also found that irritability was significantly associated with temporal reward discounting impulsivity [27] using one computer-based delayed discounting task, which is similar to the MCQ scale in this study. Differences in types of reward processing, samples, and measurements may explain the negative findings in this study. Considering that delayed reward discounting is strongly associated with anhedonia and affective disorders [62], more studies are needed to replicate our findings.

### Insomnia

As expected, insomnia was an important mediating factors between AS and irritability, which can explain 20% variation of irritable symptoms. A significant association of AS and sleep related problems has been reported in various samples of adolescents [63]. AS is characterized by heightened focus on physical sensations, cognitive concerns, and social performance. Individual with high AS may be particularly susceptible to this phenomenon at bedtime, which may delay the ability to fall asleep and disturb sleep maintenance. Further, sleep loss would cause impaired ability of sleep regulation and difficulties in impulse control [20]. When facing frustration or inappropriate reward, then these individuals tend to show irritable symptoms [18].

### Selective attention for threat

Consistent with our hypothesis, selective attention for threat also mediated the relationship between AS and irritability. AS amplifies anxiety-related fear and may lead to an abnormal sensitivity to threat in the environment [31]. Excessive attention to potentially threatening stimuli in environment may lead to a lower threshold for frustration [2]. However, the effect size of relationship between elective attention for threat and irritability was small. This may reflect some truth, as some studies reported no significant attention bias to threat among high irritability [64]. It is also may be that the self-report question used in this study can’t effectively measure attention bias.

### Limitations

The present study has several limitations that should be noted when interpreting the results. First, this study reported cross-sectional data of the project, prospective data are needed to determine the longitudinal relationships. Second, self-report questionnaires are used to measure our main outcomes, parents/teachers’ reports or interview methods should be considered to reduce bias. Finally, we only used four items from the Chinese DACOBS subscale to assess attention for threat bias. Other measures such as dot-probe task may be more effective to measure attentional bias.

## Conclusions

The findings suggest that AS is an important susceptibility factor for irritability. Insomnia and selective attention for threat are two important psychological mechanisms to understand the phenomenon. Longitudinal studies are needed to replicate our main findings and AS and related mechanisms should be considered in treatment of irritability.

## Data Availability

The raw data and analysis scripts are openly available at https://data.mendeley.com/drafts/yxbmj2b4jw.
